# Recurrent aneurysmal fibrous histiocytoma of the breast with malignant features: a rare case report

**DOI:** 10.3389/fonc.2026.1899426

**Published:** 2026-10-19

**Authors:** Kui Xiao, Nianqu Zhang, Qing Li, Qiaoran Liu

**Affiliations:** 1 Shandong First Medical University (Shandong Academy of Medical Sciences), Jinan, Shandong, China; 2 Department of General Surgery, The First Affiliated Hospital of Shandong First Medical University, Jinan, Shandong, China; 3 Key Laboratory of Laparoscopic Technology, The First Affiliated Hospital of Shandong First Medical University, Jinan, Shandong, China; 4 Key Laboratory of Metabolism and Gastrointestinal Tumor, The First Affiliated Hospital of Shandong First Medical University, Jinan, Shandong, China

**Keywords:** aneurysmal fibrous histiocytoma, breast cancer, breast neoplasm, mesenchymal tumor, undifferentiated pleomorphic sarcoma

## Abstract

Aneurysmal fibrous histiocytoma (AFH) is generally a benign soft tissue tumor that most commonly occurs in the extremities and trunk. However, its primary presentation in the breast is exceedingly rare. Within this exceptionally rare subset, only isolated case reports have described recurrent AFH with malignant features. Here, we present a case of recurrent aneurysmal fibrous histiocytoma with malignant features occurring in the breast. Following recurrence, a 73-year-old female underwent modified radical mastectomy. Based on the histopathological and immunohistochemical evaluation of the resected specimen, combined with imaging findings, a final diagnosis of undifferentiated pleomorphic sarcoma (UPS) was established. The tumor in this case exhibited an immunophenotype positive for CD34, CD63, and CD68, with a Ki-67 index of approximately 30%, and harbored BCOR and ARID1A gene mutations. We discuss the histologic, immunohistochemical, and molecular findings that supported the diagnosis of UPS and consider whether this recurrent tumor represents progression from the earlier AFH.

## Introduction

Aneurysmal fibrous histiocytoma (AFH) is a rare subtype of fibrous histiocytoma that most commonly occurs on the extremities, head, neck, and trunk (1) (in this report, AFH refers to aneurysmal fibrous histiocytoma, distinct from angiomatoid fibrous histiocytoma, an EWSR1-rearranged tumor). This entity predominantly affects middle-aged adults, with a slight female predominance (2). Its pathological features include a dermal nodule composed of spindle and pleomorphic cells, containing central hemorrhagic cystic spaces lacking endothelial lining, extravasated erythrocytes, and hemosiderin deposition (1). Aneurysmal fibrous histiocytoma is generally benign, with a relatively high local recurrence rate; in extremely rare instances, AFH may exhibit aggressive growth or even distant metastasis (2–4). When fibrous histiocytoma displays malignant features, it is currently classified within the undifferentiated pleomorphic sarcoma (UPS) category. The historical term malignant fibrous histiocytoma (MFH) has been incorporated into the UPS group in the current WHO classification (5); in this report, UPS is used except when citing historical literature. Direct clonal progression from AFH to UPS has not been molecularly demonstrated, and such cases are exceedingly rare. We report a recurrent breast AFH that at recurrence showed high-grade pleomorphic morphology and was diagnosed as UPS.

## Case report

A 73-year-old Chinese female patient first presented to the hospital in 2021 due to a painless, palpable nodule in her right breast. The nodule was initially about the size of a peanut but suddenly enlarged to the size of an egg during physical labor. Physical examination revealed a firm, well-defined, immobile mass measuring 4–5 cm, with no abnormal changes in the overlying skin.

Ultrasound detected a cystic-solid mass measuring 5.1 × 3.3 × 5.2 cm with clear boundary, irregular shape and internal vascularity (**Figure 1**). Partial mastectomy was performed to remove the nodule. Histopathological examination identified false blood vessel spaces of varying sizes within the tumor. The tumor was composed of pleomorphic cells arranged in a storiform pattern (**Figure 2a**), with significant hemosiderin deposition (**Figure 2b**). Immunohistochemical staining was positive for CD34 (**Figure 2c**), and negative for CD68, CD163, SMA and STAT6. Ki-67 index was approximately 20% (**Figure 2d**). The tumor was diagnosed as AFH. The patient was discharged without further treatment.

**Figure 1:**
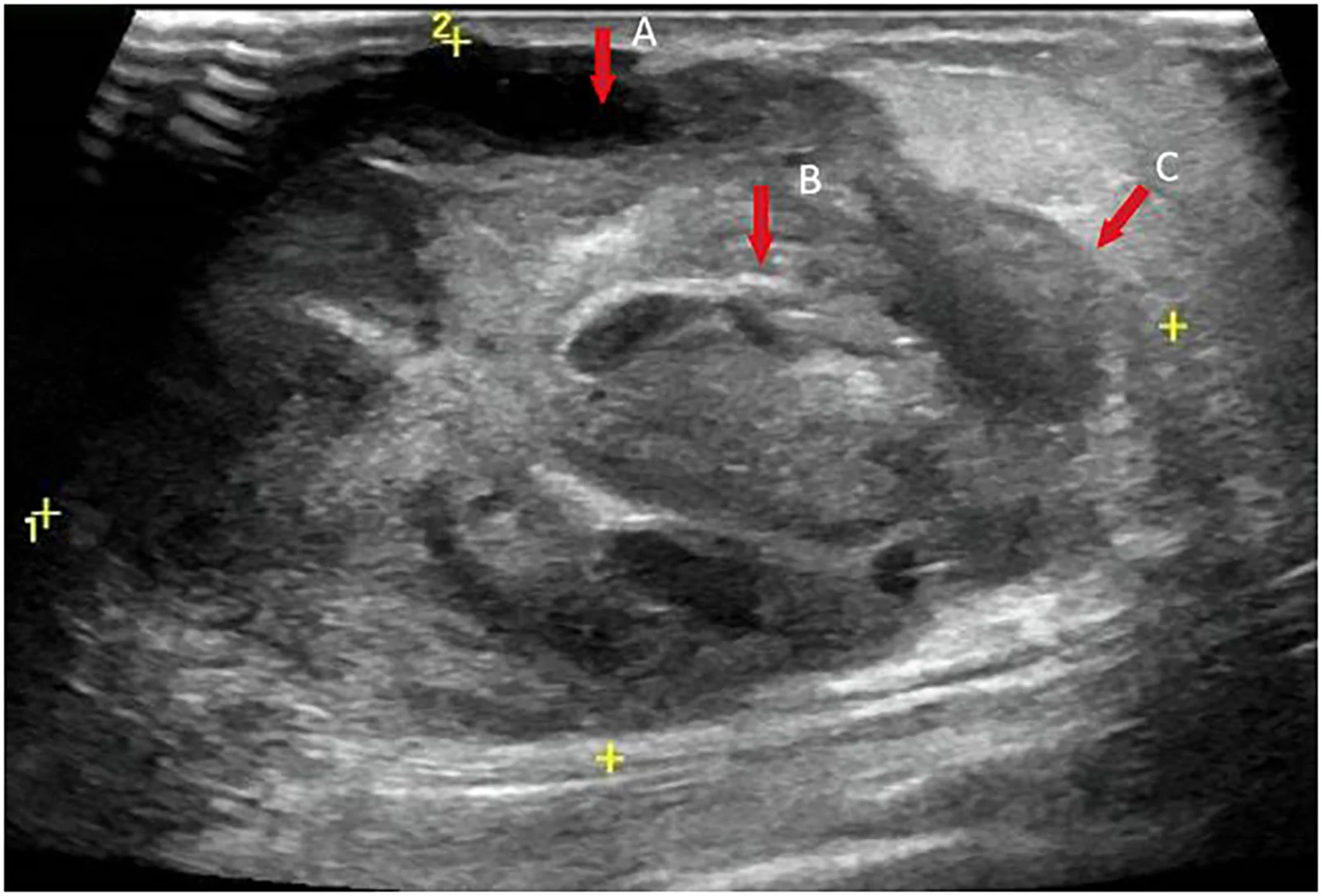
Ultrasound findings of the initial lesion. Ultrasound demonstrates a cystic‑solid mass measuring 5.1 × 3.3 × 5.2 cm. Arrow A indicates the cystic component of the mass, arrow B denotes the solid component, and arrow C marks the clear yet irregular border of the mass. Vascular signals are identified within the solid component.

**Figure 2:**
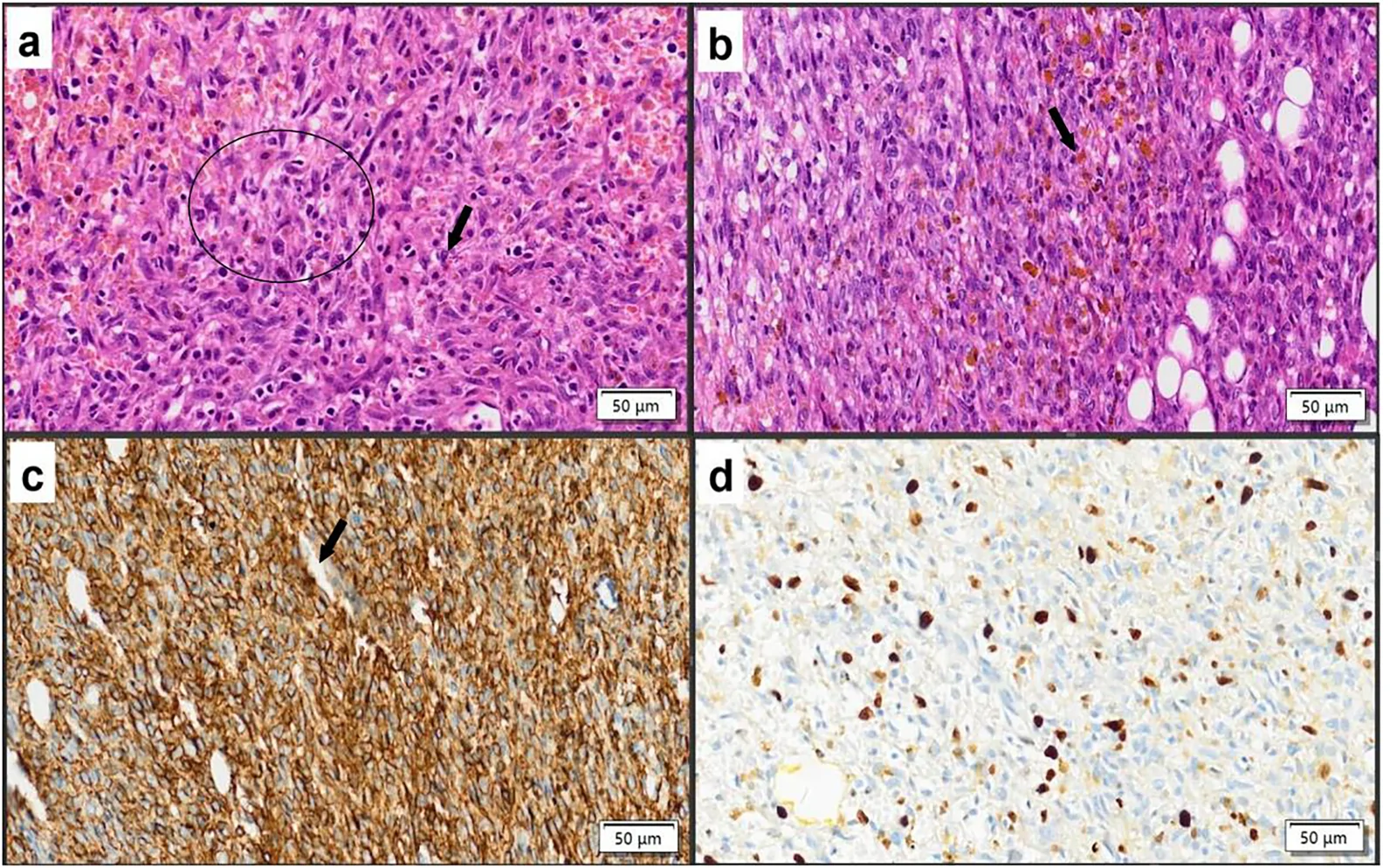
Histopathologic findings of the recurrent lesion. **(a)** H&E staining, open circle highlights the characteristic storiform growth pattern; thin arrow points to a pathological mitotic figure, showing pleomorphic tumor cells (200×). **(b)** H&E staining, thin arrow indicates hemosiderin pigment deposition in tumor stroma (200×). **(c)** Immunohistochemical staining for CD34, arrowhead marks pseudovascular space (200×). **(d)** Immunohistochemical staining for Ki-67 (200×). Scale bars = 50 μm.

In 2023, the patient experienced local recurrence. A painless 2 × 2 cm nodule was palpated at the previous surgical site of the right breast, which gradually enlarged to approximately 4 × 2 cm over four months. Mammography revealed slightly lobulated, round mass with clear boundaries (**Figure 3a**). During the examination, the patient felt a sudden pain and rapid enlargement of the tumor. Ultrasound detected a mixed echogenic mass measuring 5.8 × 2.8 cm with clear boundaries and regular morphology (**Figure 3b**). Arterial blood flow signals were detected in the solid portion of the mass.

**Figure 3:**
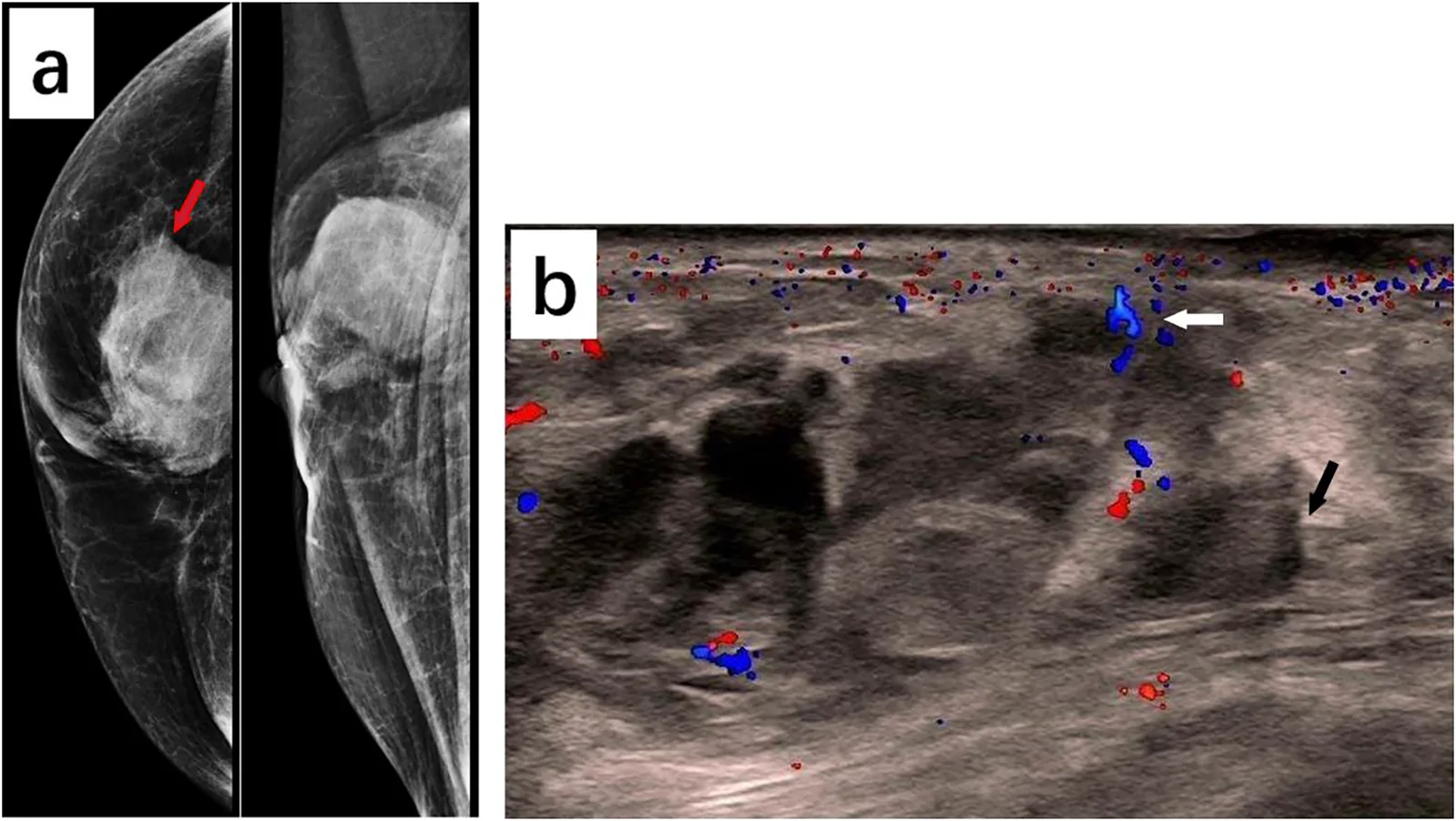
Mammography and ultrasound findings of the recurrent tumor. **(a)** Mammography: The mammogram reveals a well-defined, rounded mass with slight lobulation, indicated by the red arrow. **(b)** Ultrasound: The ultrasound image demonstrates a mixed echogenic mass measuring 5.8 × 2.8 cm, with well-circumscribed borders and regular overall morphology. Color Doppler reveals arterial flow signals within the solid components, confirming internal vascularity. The black arrow marks the tumor margin, and the white arrow points to these intralesional arterial flow signals.

Due to the tumor’s recurrence, a modified radical mastectomy and partial axillary lymph node dissection were performed. Pathological examination revealed spindle and pleomorphic cells with prominent pathological mitotic figures (**Figure 4a**). Abundant multinucleated giant cells were observed and the nucleoli were prominent (**Figure 4b**). Immunohistochemical staining was positive for CD34 (**Figure 4c**), CD63 and CD68, and negative for CK, CK5/6, ER, GATA3, p63, Desmin, CD99, ALK, SMA, SOX-10 and S-100. The Ki-67 index was approximately 30% (**Figure 4d**). **Table 1** summarizes the clinical information and treatment course of this patient. To exclude molecularly defined entities such as DFSP—and to molecularly characterize this diagnostically challenging tumor and identify potentially actionable variants, we performed next-generation sequencing (NGS: Illumina NextSeq 550/NovaSeq 6000 platform, comprehensive sequencing panel for soft tissue and bone tumors, covering 113 genes in DNA and 110 fusion genes in RNA, with an effective sequencing depth of 3502× and matched peripheral blood to exclude germline mutations) on postoperative tumor tissue. Two frameshift variants were identified in BCOR [c.2488_2489del (p.S830fs), VAF 33.48%] and ARID1A [c.6134_6135del (p.K2045fs), VAF 29.67%], both predicted to result in loss of protein function (**Table 2**), and were classified as Tier II (potentially clinically significant) according to AMP/ASCO/CAP guidelines. In addition, an in‑frame deletion in PDGFRB, c.1687_1695del (p.E563_R565del), with a variant allele frequency of 28.38%, was identified and classified as Tier III (variant of uncertain significance). No known gene fusions were detected. The tumor was microsatellite stable (MSS), with no mismatch repair deficiency, and no pathogenic germline variants were identified in cancer predisposition genes.

**Figure 4:**
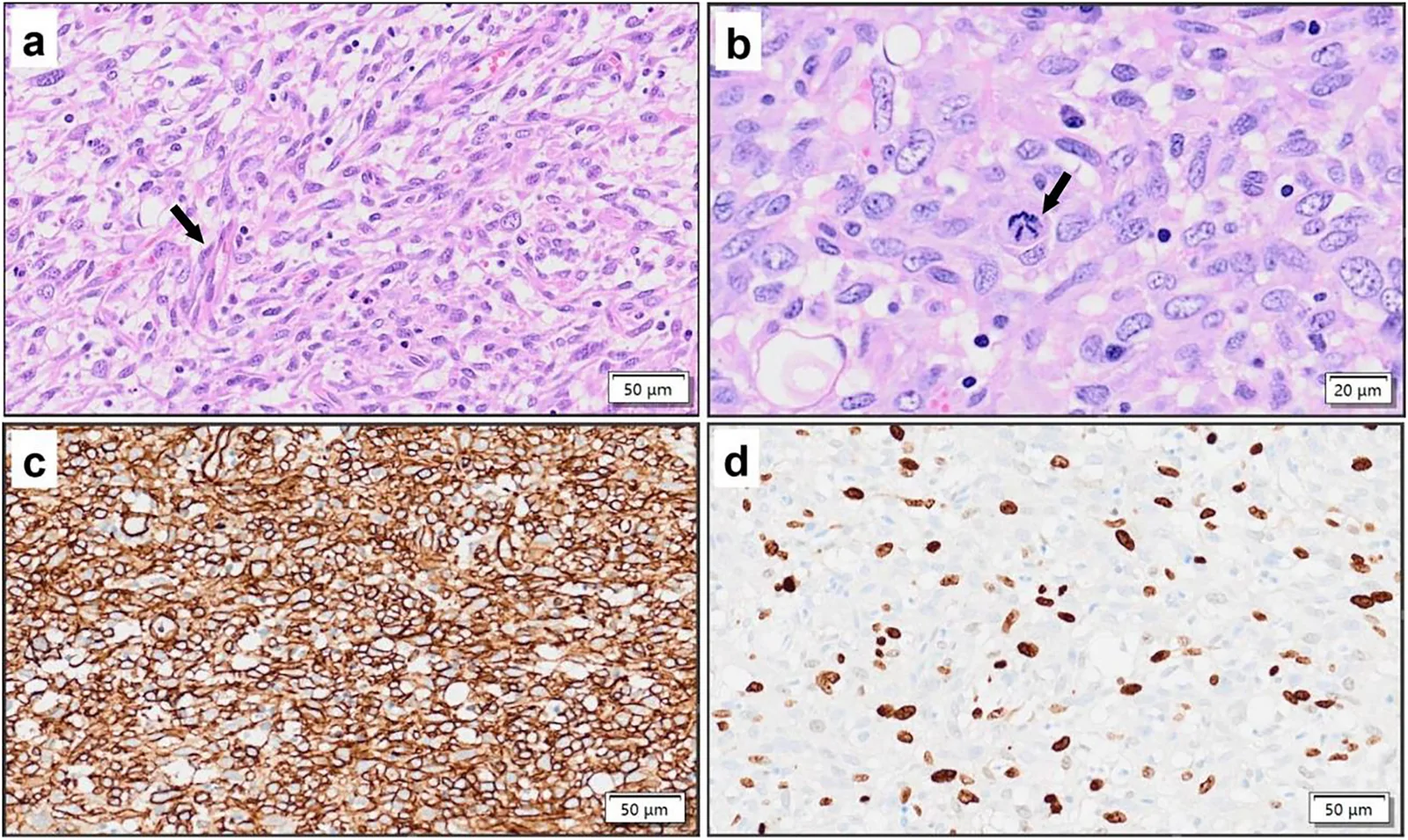
Histopathologic findings of the recurrent lesion. **(a)** Hematoxylin and eosin (H&E) staining shows proliferative spindle and pleomorphic tumor cells (black arrow; 200×). **(b)** H&E staining identifies pathological mitotic figures (black arrow; 400×). **(c)** Immunohistochemical staining for CD34 demonstrates diffuse positive expression within tumor cells (200×). **(d)** Immunohistochemical Ki-67 staining highlights proliferating tumor cell nuclei (200×).

**Table 1:** Timeline of the patient’s clinical course.

Date/Time point	Event	Key information
Phase 1: initial AFH (Benign)		
2021	Initial presentation	73-year-old female with a painless palpable nodule in the right breast, initially peanut-sized; suddenly enlarged to egg-size during physical labor
2021 (at initial presentation)	Imaging	Ultrasound: 5.1×3.3×5.2 cm cystic-solid mass, well-defined, irregular, vascular
2021	First surgery	Partial mastectomy
2021 (postoperative pathology)	Pathological diagnosis	Pathology (AFH): pseudovascular spaces, storiform pleomorphic cells, hemosiderin; IHC: CD34+, CD68–, SMA–, STAT6–; Ki-67 ~20%
Phase 2: local Recurrence with Morphologic Progression to UPS		
2023	Local recurrence	Local recurrence: 2×2 cm → ~4×2 cm over 4 months at prior site
2023 (at recurrence presentation)	Imaging	mammography – lobulated, round, clear margins; sudden pain + enlargement; ultrasound – 5.8×2.8 cm mixed echogenic mass, well-defined, regular, arterial flow
2023	Second surgery	Modified radical mastectomy + partial axillary dissection
2023 (postoperative pathology)	Pathological diagnosis	Pathology (UPS): spindle/pleomorphic cells, high mitoses, giant cells; IHC: CD34+, CD68+, CK–, p63–, Desmin–, SMA–, S100–, SOX10–; Ki-67 ~30%
2023 (postoperative)	MDT discussion	MDT: breast surgery, oncology, radiation oncology, pathology – decided against adjuvant RT/chemo
2023–2026	Follow-up	Follow-up (30 months): no local recurrence or distant metastasis

**Table 2:** Gene mutations detected by next generation sequencing.

Gene	Chromosome coordinates	Accession	Exon/intron	Nucleotide change	Amino acid change	Variant frequency
BCOR	chrX:39932110	NM_017745.6	exon4	c.2488_2489del	p.S830fs	33.48%
ARID1A	chr1:27106522	NM_006015.6	exon20	c.6134_6135del	p.K2045fs	29.67%

Based on the patient’s history and tumor characteristics, the recurrent lesion was diagnosed as undifferentiated pleomorphic sarcoma (UPS). The maximum tumor diameter was 5.0 cm (measured as 5×4×2 cm). The closest distance to the skin was 0.8 cm, and the closest distance to the deep resection margin was 0.2 cm. The pathological report clearly stated “no involvement of nipple, skin, or deep resection margin,” indicating negative surgical margins (R0 resection). Thirteen axillary lymph nodes were identified, all negative for tumor cells (pN0). The recurrent tumor was classified as FNCLCC grade 2. As an undifferentiated sarcoma, the differentiation score was 3, and no tumor necrosis was identified. The pathology report recorded 7–10 mitoses per 10 HPFs, a range spanning the threshold between mitotic scores 1 and 2; in either case the overall FNCLCC grade remained G2.

Postoperative gross pathological examination demonstrated the tumor’s maximum diameter of 5.0 cm (5×4×2 cm). Notably, preoperative ultrasound measured the lesion up to 5.8 cm, whereas non-contrast CT recorded 4.5 cm. This discrepancy between imaging and pathological tumor dimensions can be attributed to peritumoral edema visualized on ultrasound and physiological shrinkage of the tumor following surgical resection. Given the absence of regional lymph node metastasis (N0) and no evidence of distant metastasis (M0), the final AJCC staging (8th edition for soft tissue sarcoma) was pT1N0M0G2, Stage IIA.

After multidisciplinary team (MDT) discussion involving breast surgery, medical oncology, radiation oncology, and pathology, the patient did not receive adjuvant radiotherapy or chemotherapy. This decision was individualized, taking into account her advanced age (73 years), R0 resection with negative margins, the uncertain applicability of evidence derived mainly from extremity and trunk soft tissue sarcomas to primary breast UPS, the potential cardiopulmonary and soft-tissue toxicity of breast-region radiotherapy, and the patient’s preference. Close follow-up is ongoing. As of the last follow-up at 30 months postoperatively, the patient remains free of local recurrence or distant metastasis.

## Discussion

Aneurysmal fibrous histiocytoma is a rare tumor that predominantly occurs in the extremities, head and neck, and trunk. It is characterized by fibrohistiocytic cell proliferation containing hemorrhagic cystic spaces lacking endothelial lining (1, 2). Although aneurysmal fibrous histiocytoma is generally benign, it has a high recurrence rate and may occasionally undergo malignant transformation or develop regional/distant metastases (2–4). This case report describes an aneurysmal fibrous histiocytoma arising in the breast that recurred locally, with the recurrent lesion showing malignant histopathological features and subsequently diagnosed as undifferentiated pleomorphic sarcoma (UPS), without evidence of metastasis. When aneurysmal fibrous histiocytoma involves the skin, it presents as blue, black, or red nodules (1). However, in this patient, the aneurysmal fibrous histiocytoma was located deep in the breast tissue without skin involvement. The patient experienced rapid enlargement of the mass at both the first and second occurrences, which is consistent with the characteristic abundant vascularity of aneurysmal fibrous histiocytoma and the possibility of acute hemorrhage that can lead to sudden tumor enlargement. At recurrence, the lesion showed marked cytological pleomorphism and increased mitotic activity and was subsequently diagnosed as undifferentiated pleomorphic sarcoma (UPS). UPS is divided into five main subtypes: storiform-pleomorphic, myxoid, giant cell, inflammatory, and angiomatoid (6). Since 2020, the World Health Organization (WHO) has incorporated the previously designated malignant fibrous histiocytoma (MFH) into the UPS group (5). UPS is a rare sarcoma subtype in the breast, and cases of UPS arising after recurrence of aneurysmal fibrous histiocytoma of the breast are even rarer.

In the present case, the recurrent lesion was diagnosed as undifferentiated pleomorphic sarcoma (UPS) based on its histopathological and immunohistochemical features, together with the clinical history of a previously diagnosed aneurysmal fibrous histiocytoma. Since UPS is essentially a diagnosis of exclusion, which can only be rendered after ruling out all other entities with definable differentiation, a systematic differential diagnostic analysis was performed in this study.

The priority diagnostic consideration in this case was malignant phyllodes tumor, which had to be excluded. The patient was a 73-year-old female presenting with a rapidly growing cystic-solid mass that recurred postoperatively and was CD34-positive—clinicopathological features that significantly overlap with malignant phyllodes tumor (7). Even so, histologically, malignant phyllodes tumor is characterized by a characteristic leaf-like structure, consisting of hyperplastic stromal cells surrounding slit-like spaces lined by biphasic epithelial cells (7, 8), and in this case, no typical leaf-like cleft structures were observed, nor was there any residual epithelial component engulfed by stroma, which constitutes the primary morphological basis for excluding this diagnosis (7, 8). From an immunophenotypic perspective, CD34 expression in breast phyllodes tumors is inversely correlated with tumor grade, with its positivity rate decreasing significantly as the grade increases. Multiple studies have reported CD34 positivity rates of 38.3%–100%, 33.3%–75%, and 5.9%–57% in benign, borderline, and malignant phyllodes tumors, respectively (9–11); expression is commonly reduced or lost in high-grade lesions (12). In our case, CD34 showed diffuse and strong positivity, which is clearly inconsistent with the typical pattern of reduced CD34 expression in malignant phyllodes tumors. Furthermore, although stromal cells in malignant phyllodes tumors may show focal positivity for broad-spectrum cytokeratins and p63 (13), the epithelial markers in this case were all negative, further supporting the exclusion of a malignant phyllodes tumor diagnosis (13).

A second diagnostic possibility was spindle cell metaplastic carcinoma, another important consideration in the workup of primary spindle cell lesions of the breast (14). The diagnosis of spindle cell metaplastic carcinoma relies on evidence of epithelial differentiation, with a characteristic immunophenotype showing positivity for broad-spectrum cytokeratins (CKpan/AE1/AE3), high-molecular-weight cytokeratins (CK5/6, 34βE12), and p63 (15, 16). In the tumor cells, all of these epithelial markers were negative, directly ruling out a tumor of epithelial origin. Of note, although stromal cells in some malignant phyllodes tumors may show focal positivity for CK or p63 (13), the complete absence of all epithelial markers in this case is sufficient to exclude the diagnosis of spindle cell metaplastic carcinoma.

Given the diffuse and strong CD34 positivity, dermatofibrosarcoma protuberans (DFSP) and its fibrosarcomatous variant were considered in the differential diagnosis (17). Nonetheless, CD34 positivity alone is not diagnostic. DFSP was excluded after integrating the morphologic, immunophenotypic, and molecular findings. DFSP is characterized by the COL1A1::PDGFB gene fusion, which is detected in 91.4% to 96% of DFSP cases (18, 19). In our case, fluorescence *in situ* hybridization (FISH) for the COL1A1::PDGFB fusion was negative, thereby unequivocally excluding DFSP (20). With respect to ancillary immunohistochemistry, factor XIIIa staining is typically negative in DFSP, and our case also showed negative staining for factor XIIIa, which is consistent with the DFSP phenotype; but the negative FISH result provides the most reliable evidence for exclusion (21). Histologically, classic DFSP exhibits a storiform arrangement of spindle cells with honeycomb-like infiltration into the subcutaneous fat (17). Although spindle cells were present in our case, the characteristic storiform pattern and subcutaneous honeycomb infiltration were absent, further supporting the exclusion of this diagnosis.

In addition, other primary breast sarcomas must be excluded. Leiomyosarcoma is characterized by diffuse desmin positivity and SMA positivity on immunophenotyping (22); both markers were negative in this case, thus ruling it out. The key differential feature of angiosarcoma is positivity for CD31 and ERG (23), both of which were also negative in our case, excluding a vascular tumor. Other specific sarcomas, such as rhabdomyosarcoma (MyoD1- and myogenin-positive) and liposarcoma (MDM2- and CDK4-positive), were excluded sequentially, as our case lacked expression of the corresponding differentiation markers (24, 25).

After these exclusions, UPS was favored, and the diagnosis was established based on the combined histological and immunohistochemical findings (26). The tumor exhibited histological features consistent with a high-grade sarcoma, including pleomorphic spindle cells and increased mitotic activity (7–10 mitoses per 10 HPFs). Immunohistochemically, vimentin was diffusely positive, while epithelial markers (CK, p63, CK5/6), myogenic markers (desmin, SMA), vascular endothelial markers (CD31, ERG), and melanoma markers (S100, HMB45, SOX10) were all negative. Although focal, patchy SMA positivity may be observed in UPS, it is not regarded as evidence of specific differentiation; only diffuse and strong positivity would be suggestive of a specific line of differentiation (27).

Histologically, both AFH and UPS exhibit spindle and pleomorphic cells arranged in a storiform pattern. The typical immunophenotype of aneurysmal fibrous histiocytoma is vimentin positivity, with negativity for CD31, CD34, desmin, and factor XIIIa (2). Negative CD34 staining in tumor cells surrounding the vascular spaces helps rule out a vascular endothelial origin (28). However, rare individual case reports have described focal CD34 positivity in AFH tumor cells (29), suggesting that CD34 negativity is not absolute. Moreover, UPS, as a high-grade sarcoma, lacks a specific immunophenotype (27), which further increases the diagnostic difficulty.

The uniqueness of the present case lies in the fact that both the primary AFH component and the recurrent UPS component retained CD34 positivity. In light of the rare CD34-positive AFH cases reported in the literature (29), this may suggest that the aneurysmal fibrous histiocytoma in this case belongs to a rare CD34-positive variant subtype, and that CD34 expression was retained during recurrence or progression. Whether this represents true malignant transformation remains speculative. Considering that CD34 expression is non-specific, CD34 positivity alone cannot confirm a distinct subtype or malignant transformation. In the differential diagnosis, CD34-positive mammary AFH should be distinguished from other CD34-positive spindle cell lesions of the breast, such as solitary fibrous tumor (SFT) (30, 31) and dermatofibrosarcoma protuberans (DFSP) (17, 19). Interpretation should be integrated with morphology, immunophenotype, molecular findings, and clinical history.

The prognostic significance of CD34 in UPS has been supported by evidence-based medicine—a study of 192 soft tissue sarcomas, predominantly myxofibrosarcomas, demonstrated that CD34-positive patients had significantly better overall survival and distant metastasis-free survival than CD34-negative patients (Cox model hazard ratio = 3.327; 95% confidence interval 1.334–8.295), suggesting that CD34 expression status may serve as an independent prognostic factor in this series (32). Although this study primarily focused on myxofibrosarcomas, its findings remain informative for the prognostic assessment of UPS. In the present case, tumor cells in both the AFH and UPS phases retained CD34 expression, which, based on the above evidence, may suggest that its biological aggressiveness is lower than that of CD34-negative UPS. However, CD34 positivity is hypothesis-generating and should not currently be used to guide treatment, since this inference still requires validation in additional similar cases.

Molecular profiling is increasingly integrated into the diagnostic work-up of mesenchymal tumors (33). Because sarcomas often overlap in morphology and immunophenotype, genomic data can supplement conventional pathology, refine the diagnosis, and add biological information, particularly in challenging cases (34). The Italian Sarcoma Group consensus emphasizes that extended molecular profiling can refine diagnosis, improve patient stratification, and guide treatment even when no direct target is found, and that molecular tumor boards are important for optimal interpretation (33). Patton and Dermawan similarly note that fusion detection and targeted sequencing are improving diagnostic accuracy in soft tissue sarcoma and helping to develop targeted therapies (34). In our patient, molecular testing helped exclude definable sarcoma entities and provided additional molecular characterization, but it did not establish clonal evolution from AFH to UPS. With the continuous advancement of molecular pathology, some cases previously diagnosed as UPS have been reclassified as other specific sarcomas upon more comprehensive testing (31, 35). UPS diagnosis therefore still requires a broad immunohistochemical panel; when feasible, molecular testing should also be used to exclude specific gene fusions or amplifications (27, 36). In this case, next-generation sequencing (NGS) did not detect any known gene fusions but identified frameshift variants in ARID1A, c.6134_6135del (p.K2045fs), and BCOR, c.2488_2489del (p.S830fs). The corresponding variant allele frequencies were 29.67% and 33.48%, respectively. These findings provide additional molecular characterization of the recurrent tumor. In the clinical NGS report, both frameshift variants were classified as Tier II (potentially clinically significant) under the AMP/ASCO/CAP tiering system. Because both are predicted to cause loss of protein function, we also assessed their potential pathogenicity in a research setting using ACMG/AMP criteria.

ARID1A encodes a core subunit of the SWI/SNF chromatin remodeling complex, and is recognized as a classic tumor suppressor gene (37). Its loss of function is extremely common in epithelial tumors (mutation frequencies: 8%–60%), but rare in sarcomas—a NGS study of 584 soft tissue sarcomas identified only 4 cases (0.7%) (37). In our case, the ARID1A c.6134_6135del (p.K2045fs) frameshift mutation was identified. This variant meets the PVS1 criterion (37, 38), is absent from gnomAD and ClinVar and thus meets PM2 (39), and is supported by PP4 because the patient’s UPS phenotype is consistent with the ARID1A loss-of-function tumor spectrum (37). Integrating these lines of evidence, the variant was classified as “Pathogenic” (38, 39). Despite this, this classification only indicates that the variant is likely to impair ARID1A function. It does not prove that it drove the AFH-to-UPS transformation.

BCOR encodes the BCL-6 corepressor and participates in epigenetic regulation of gene transcription (40). BCOR abnormalities have been reported to manifest as gene fusions (e.g., BCOR::CCNB3) and internal tandem duplications (ITD) (40, 41), as well as loss-of-function (LoF) point mutations or frameshift mutations (42). In this case, no BCOR fusion was detected, instead, a c.2488_2489del (p.S830fs) frameshift mutation was found. This variant meets ACMG/AMP criteria PVS1 and PM2 (38, 43), and was classified as “Pathogenic” (38). As with ARID1A, this finding points to a possible contribution of BCOR loss of function to the biology of this tumor. However, no functional studies have yet demonstrated a driver role for it in UPS.

In this case, dual pathogenic frameshift mutations in ARID1A and BCOR were detected, providing insights into the molecular pathogenesis of this tumor and the high genetic heterogeneity of UPS (40, 42, 44). But they are not specific alterations in AFH/UPS, and the specific interaction and causal relationship between the two in this case currently lack direct functional validation and lineage-tracing evidence, and require further investigation.

UPS accounts for approximately 15% of all soft-tissue sarcoma cases and has a 5-year survival prognosis of around 60% (45). Complete surgical resection with negative margins remains the cornerstone of treatment for localized UPS. Adjuvant radiotherapy is generally considered on an individualized basis according to tumor grade, size, anatomic site, and margin status (46). The NCCN guidelines state that adjuvant radiotherapy should not be routinely administered and should be reserved for highly selected patients, and only when local recurrence would cause substantial morbidity (46). In extremity and trunk soft tissue sarcomas, adjuvant radiotherapy after R0 resection may improve local control, but an overall survival benefit has not been established, and the evidence is conflicting (47, 48). Moreover, these data are derived mainly from extremity/trunk cohorts, and their applicability to primary breast UPS remains uncertain (48). Evidence for radiotherapy in primary breast sarcoma is even more limited, and current recommendations largely pertain to high-risk features such as large tumor size, close/positive margins, or recurrence (48). In the present case, the recurrent tumor was completely resected with negative margins, but the closest deep margin was 0.2 cm, which constitutes a close margin, indicating a non-negligible risk of local recurrence. Therefore, whether to proceed with adjuvant radiotherapy became a matter of debate.

After MDT discussion, adjuvant radiotherapy was ultimately omitted based on a composite consideration of the patient’s advanced age (73 years), the potential cardiopulmonary and soft-tissue toxicity of breast-region radiotherapy, the uncertainty of extrapolating extremity/trunk evidence to breast UPS, and the patient’s own preference. Although comprehensive treatment including radiotherapy may improve outcomes in elderly patients with STS, it requires caution because of increased age-related toxicity (49). This decision should be regarded as an individualized multidisciplinary clinical decision for this elderly patient, rather than a conclusion that the available evidence clearly supports omitting radiotherapy in similar cases (50). If local recurrence occurs, salvage treatment including re-excision or radiotherapy could still be considered. The patient remains under close follow-up, with no local recurrence or distant metastasis at 30 months postoperatively. The necessity of adjuvant chemotherapy and radiotherapy warrants further investigation (43, 47).

## Data Availability

The datasets presented in this study are not publicly available because their access is restricted by the Regulations on the Management of Human Genetic Resources of the People's Republic of China. The processed sequencing evaluation data have been deposited in Zenodo under https://doi.org/10.5281/zenodo.23149562. Requests to access the raw sequencing data should be directed to the corresponding author and are subject to a material transfer agreement.
